# Dynamics from elastic neutron-scattering via direct measurement of the running time-integral of the van Hove distribution function

**DOI:** 10.1038/s41598-019-46835-z

**Published:** 2019-08-02

**Authors:** Antonio Benedetto, Gordon J. Kearley

**Affiliations:** 1 School of Physics, University College Dublin, Dublin 4, Ireland; 2 School of Chemistry, University College Dublin, Dublin 4, Ireland; 3 Conway Institute of Biomolecular and Biomedical Research, University College Dublin, Dublin 4, Ireland; 4 Department of Sciences, University of Roma Tre, Rome, Italy; 5 Laboratory for Neutron Scattering, Paul Scherrer Institut, Villigen, Switzerland

**Keywords:** Chemical physics, Techniques and instrumentation

## Abstract

We present a new neutron-scattering approach to access the van Hove distribution function directly in the time domain, I(t), which reflects the system dynamics. Currently, I(t) is essentially determined from neutron energy-exchange. Our method consists of the straightforward measurement of the running time-integral of I(t), by computing the portion of scattered neutrons corresponding to species at rest within a time *t*, (conceptually elastic scattering). Previous attempts failed to recognise this connection. Starting from a theoretical standpoint, a practical realisation is assessed via numerical methods and an instrument simulation.

## Introduction

In this letter we present a new method for probing single-particle dynamics, which are central to the study of atomic and molecular motion across many areas of research. These dynamics can be fully described by the van Hove self-distribution function, G_Self_(r,t), which represents the probability that a species has diffused a distance *r* over time *t*. Equivalently, by its spatial Fourier transform, I(Q,t), which represents the probability that a species is within a volume 4/3π(2π/Q)^3^ after a time *t*, where ħQ = 2πħ/r is the momentum transfer^1^. For our discussion we ignore the Q-dependence of the function, but for generality we retain “van Hove” to denote I(t). Probing these dynamics at molecular or atomic scales requires the use of techniques such as X-ray and neutron scattering. Neutrons are unique in being scattered by the atomic nuclei, rather than measuring the response of the electron clouds to the nuclear motion, as is the case in most other spectroscopies. This simplifies the analysis, and allows scattering contrast to be varied by isotopic composition, especially hydrogen and deuterium that are particularly important in polymers, solvation, soft matter systems and biosystems.

Current neutron-scattering spectroscopies for measuring these dynamics rely on the exchanged energy of each scattered neutron as shown by Brockhouse^2^. By scanning the energy difference between the neutron beam incident on the sample and that scattered by the sample, the scan is made with respect to one of the energies, the other energy being fixed^3–5^. Neutron spin-echo is different, but still encodes exchanged energy, here via neutron spin, providing I(t)^6^, whereas other methods only provide S(ΔE), the Fourier transform of I(t), where ΔE is the neutron-energy exchange.

Rather than these “inverse” approaches, we propose an approach that uses the proportion of neutrons scattered elastically within different observation times, *t*_*obs*_ (i.e. different energy resolutions), that is the probability that particles are stationary (within the time resolution of the measurement) after time *t* = *t*_*obs*_, to access I(t) directly. This is fundamentally different, and our contention is that direct access to the van Hove I(t) function should have significant advantages in instrument design, and data analysis, in particular for soft matter systems and biosystems of high (structural and dynamical) complexity.

**Figure 1:**
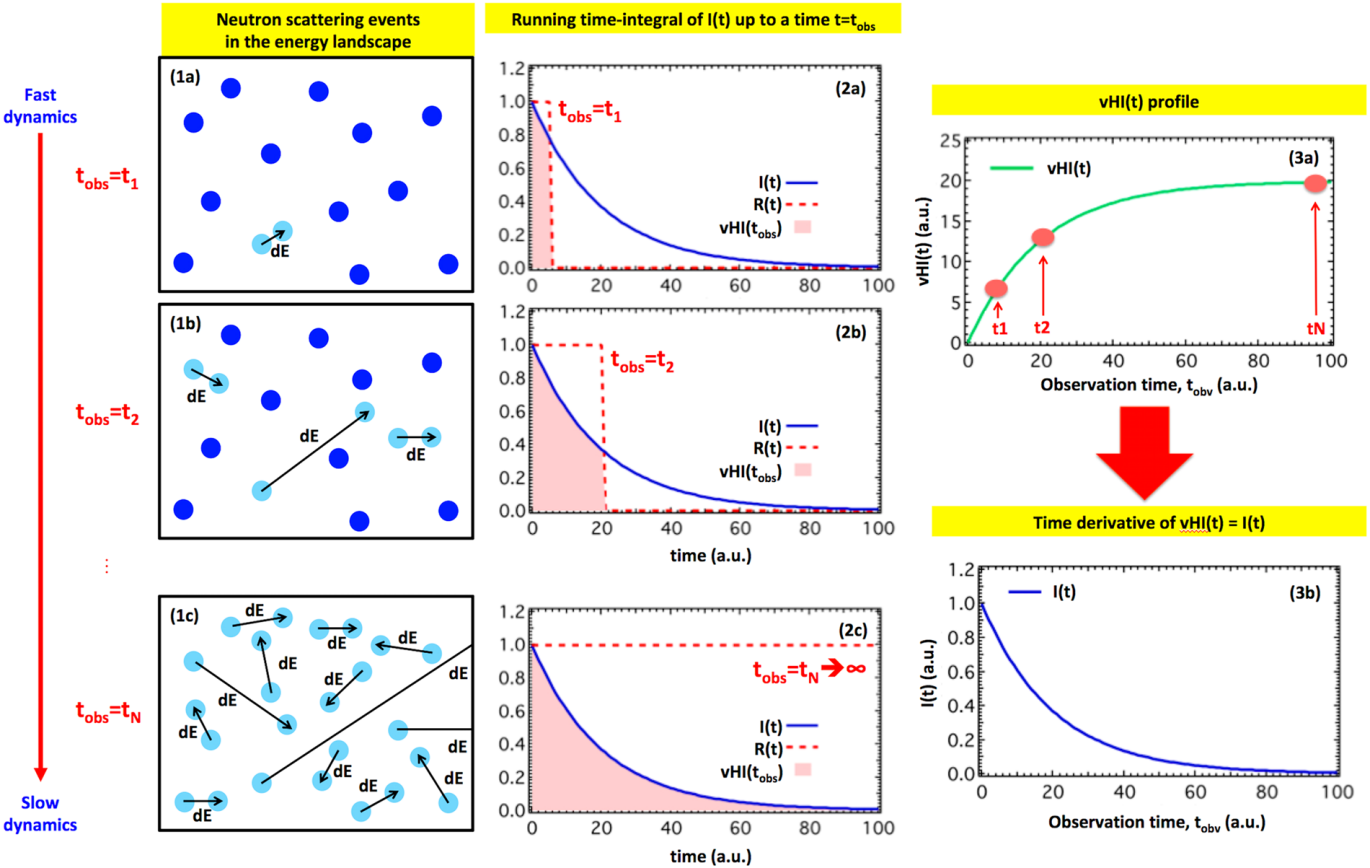
Illustration of the concept. Column 1 sketches the energy landscape of a system of particles, at three different observation times, *t*_*obs*_. *t*_*obs*_ is the time-resolution of the measurement and is inversely proportional to the instrumental energy-resolution. At short *t*_*obs*_ only the rapid motions are detected, most of the system appearing at rest (1a). At intermediate *t*_*obs*_ other motions are detected (1b), and for long *t*_*obs*_ the slower motions are also detected (1c). Existing techniques require determination of many exchanged energy values, ΔE, to access I(t), typically operating at fixed *t*_*obs*_. In general, our approach of obtaining the proportion of particles “at rest”, as a function of *t*_*obs*_, should be more efficient. This proportion formally corresponds to the running time-integral of I(t) of Eq. 2, that is the van Hove integral vHI(t = *t*_*obs*_), as sketched in column 2, in which each *t*_*obs*_ determines the upper integration limit of I(t). Differentiation of the measured vHI(t) (3a) provides I(t) directly (3b).

We will show that although measurement of I(t) may be difficult, measurement of its running time-integral up to time *t* = *t*_*obs*_ (that we will denote the van Hove integral vHI(t)), is surprisingly straightforward. Figure 1 gives a schematic illustration of the concept, and provided that a suitable route to the derivative of the vHI(t) is used, I(t) is obtained without fitting, modeling or Fourier transform.

This letter first presents the underlying theory, and then supports this by a numerical simulation. Counting-errors are then included to approach the real experiment, and finally an assessment using a Monte-Carlo instrument simulation illustrates one possible realisation of an instrument. The supplementary materials (SM) provides more detail of the theoretical approach, the protocol to extract I(t) from the measured vHI(t), and the instrument design.

## The State-of-the-Art in “Elastic Neutron Scattering for Dynamics”

Before introducing our theoretical framework, we will outline here related earlier work that tried to connect “elastic scattering” to I(t) and to system dynamics in general.

To the best of our knowledge the first attempt was made by Doster and co-workers in 2001^7–9^. These authors suggested the measured profile of elastic scattering vs energy resolution to be equal to I(t).

A second attempt was made in 2011^10,11^ in which other authors show that an inflection point occurs in the (lin-log) plot of elastic neutron scattered intensity vs energy resolution at the point where the instrumental observation time matches the system overall relaxation time. This approach has been referred to as Resolution Elastic Neutron Scattering (RENS). It was clear that the RENS spectrum was not I(t), and consequently it was shown that the approach of Doster and co-workers (above mentioned) is conceptually incorrect.

Later in 2016^12^ we proposed two new instrument concepts (suitable, respectively, for continuous and pulsed sources) designed with the specific purpose of measuring the RENS profile directly, i.e. elastic intensity vs energy resolution. This instrument would enable the overall relaxation time to be measured without Fourier transform or modeling. However, the instrument layouts, imposed a mis-match between the resolutions of the primary and secondary spectrometers. It turns out that having one of these resolutions much finer than the other is exactly what is required to access I(t) from the elastic intensity vs energy resolution. This forms the central part of this Letter, and we call this new spectroscopy for dynamics “Elastic Scattering Spectroscopy” (ESS) to distinguish from RENS and the other previous attempts.

Over the last decade, other experimental works used “elastic scattering” to access the system dynamics^13–15^, also with the support of computations^16,17^.

## The New Theoretical Framework

The starting point in the new theoretical approach we propose is to quantify “what is actually measured”, i.e. the number of neutrons counted at the detectors, in these general types of neutron-scattering experiments. In doing so, instrumental features that contribute to the neutron-energy uncertainties are naturally included in the theory.

In this new framework, the number of elastically-scattered neutrons counted at a specific instrumental condition is a definite time-integral containing the van Hove I(t) function (see SM Paragraphs 1 and 2 for more detail):

1 $${N}_{neutron}(Q,{\rm{\Delta }}E=0)={\int }_{0}^{\infty }I(Q,t)R(t;{\omega }_{R},{\rm{\Delta }}{\omega }_{R})F(t;{\omega }_{R},{\rm{\Delta }}{\omega }_{F})dt$$

Equation 1 is quite general because it is the product of:

- the incident beam, R, the incoming distribution with average energy, ħω_R_, and uncertainty, ħΔω_R_;
- the sample, I, which is the van Hove function;
- the filter analyser, F, the probability that the average energy ħω_R_ passes (where ħΔω_F_ is the width of the distribution).

All functions are properly normalized (see SM Paragraph 1 for details).

The incident-beam function, R, convolutes with the exchange processes of the sample. Normally, neutron spectroscopies then scan this energy-exchange by varying ω_R_ either in R or in F, resulting in a double convolution. In our case however, we operate entirely in the elastic regime, that is keeping fixed ω_R_, and measure vHI(t) by changing either ħΔω_R_ or ħΔω_F_, and the second convolution is absent.

For a fixed instrumental condition, Eq. 1 is the “elastic scattering”, which has been routinely measured as a function of system parameters such as temperature, pressure, hydration, etc^13,18–25,26^. In the present case however, we change the width of either the incident-beam, ħΔω_R_, or the filter, ħΔω_F_, by values that provide an incremental change in the observation time, *t*_*obs*_, that is inversely proportional to the varied (and broader) energy-width, i.e. t_obs_ = 1.66/Δω (see SM Paragraph 2.3). When the fixed width is much less than the varied width, the measurement provides vHI(t) at incremental *t*_*obs*_ times between t_min_ and t_max_, corresponding to the broadest and narrowest varied energy-widths, i.e. t_min_ = 1.66/Δω_max_ and t_max_ = 1.66/Δω_min_. It is conceptually easier to consider the case in which the incident-beam width is varied, and the analyser width is fixed (Eq. 2), although Eq. 1 shows the inverse approach to be equally valid:

2 $${N}_{neutron}(Q,{\rm{\Delta }}E=0)={\int }_{0}^{\infty }I(Q,t)R(t;{\omega }_{R},{\rm{\Delta }}{\omega }_{R})dt\approx {\int }_{0}^{{t}_{obs}=1.66/\Delta {\omega }_{R}}I(Q,t)dt\equiv vHI({t}_{obs})$$

Ideally, vHI(t) is made using a step function for R (Eq. 2). The time integral of Eq. 2 then runs from *t* = 0 to *t* = *t*_*obs*_, giving vHI(*t*_*obs*_).

## Numerical Validation

The practical R- and F-functions, however, are more likely to be Gaussian, and a crucial question that we address here is how this affects the applicability of Eq. 1. To do so, we start with a numerical simulation of the experiment in which we successively evaluate Eq. 3 (which is equivalent to Eq. 1, see SM) over a range of *t*_*obs*_ to reproduce an input function from the time domain.

3 $${N}_{neutron}(Q,{\rm{\Delta }}E=0)=\frac{{\int }_{-\infty }^{\infty }[S(Q,{\rm{\Delta }}E)\otimes R(\omega ;{\omega }_{R},{\rm{\Delta }}{\omega }_{R})]\cdot F(\omega ;{\omega }_{R},{\rm{\Delta }}{\omega }_{F})d\omega }{{\int }_{-\infty }^{\infty }S(Q,{\rm{\Delta }}E)d({\rm{\Delta }}E)\cdot {\int }_{-\infty }^{\infty }F(\omega ;{\omega }_{R},{\rm{\Delta }}{\omega }_{F})d\omega }$$

The first term of Eq. 3 is obtained from a chosen input I(t) function which is numerically Fourier transformed to the energy domain, S, and numerically convoluted with a (Gaussian) incident-beam function, R, which represents a primary monochromation device. The resulting function is multiplied with a much narrower Gaussian function, F, which represents a band-pass filter centred around the zero energy-transfer. All functions were normalised. The resulting integral, N(Q, ΔE = 0), is stored as the value of the vHI(t = *t*_*obs*_). vHI(t) is obtained via a step-wise change of *t*_*obs*_. Because we can achieve this without counting errors, we can differentiate vHI(t) numerically and compare this result with the initial I(t) input function.

**Figure 2:**
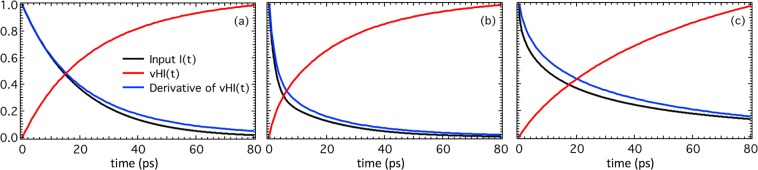
Numerical validations. The three plots are: (**a**) single exponential (with tau = 20 ps), (**b**) double exponential (with tau_1_ = 20 ps, and tau_2_ is twice as intense with = 2 ps), and (**c**) stretched exponential (with tau = 20 ps, and beta = 0.6). The other relevant parameters are: 20 < ħΔω_R_ < 15000 micro-eV corresponding to 0.127 < t_obs_ < 83 picoseconds; ħΔω_F_ = 10 micro-eV. There are no counting errors, which allows the consequences of using a Gaussian for R and F to be assessed.

The comparison has been made for three representative input functions: (i) single exponential decay, (ii) sums of exponentials, and a (iii) stretched exponential. These describe: (i) a simple isotropic translational diffusion motion, (ii) a combination of two distinct isotropic relaxations, and (iii) a continuous distribution of relaxation processes (e.g. as in complex systems like proteins), respectively. Because any dynamical relaxation, including the one described by a stretched exponential, can be expanded in a sum of single relaxations, our chosen three cases offer an almost complete picture of any potential real case. Figure 2 shows that the agreement between the input and computed functions is good overall for all these three cases. The differences originate by having as the R-function a Gaussian function rather that a step function. We would contest that for investigations on real systems, the errors entailed by the non-ideal R- and F-functions would not significantly affect the conclusions. Moreover, if required, these errors could be estimated or possibly calibrated out (e.g. by numerically computing Eq. (2) for the specific case).

**Figure 3:**
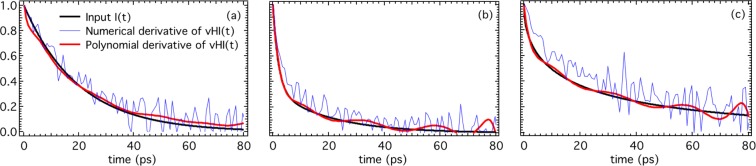
Numerical validation with counting error (described in the text). The three plots are: (**a**) single exponential, (**b**) double exponential, and (**c**) stretched exponential. The numerical derivative of vHI(t) is intractable, but its polynomial derivative reproduces the input function well. Ranges are as in Fig. 2.

To approach a real experiment, we have introduced counting errors on vHI(t). Based on the count-rate for a single point in a fixed-window scan on existing spectrometers^13^ we estimate that an integral of 10^6^ counts for the whole energy spectrum, S, would correspond to about 3 minutes per vHI(*t*_*obs*_)-point. The transmission of the final-energy filter is only ~5%. Figure 3 shows the results for the same three representative input functions of Fig. 2, illustrating that where counting errors are significant, the numerical derivative of the vHI(t) can become intractable. The problem of obtaining the derivative of noisy data is well known, and a number of solutions is available. We will consider the special case of polynomials, and then in the SM a simple Gaussian error-reduction method. Here, the polynomial approach not only provides a convenient route to the derivative, but is also physically meaningful.

**Figure 4:**
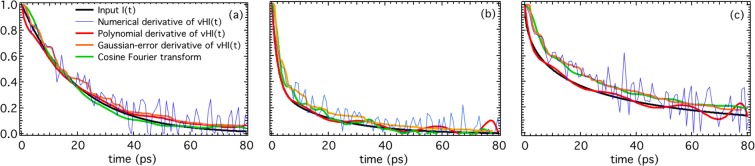
Numerical validation with counting error of several approaches to obtain I(t) from the “experimental” vHI(t). The three plots are: (**a**) single exponential, (**b**) double exponential, and (**c**) stretched exponential. The numerical derivative of vHI(t) is intractable, but its polynomial derivative and Gaussian-error derivative reproduce the input function well. The cosine FT is also shown for a consistency check. This figure is supposed for extending the results shown in Fig. 3 by showing that several approaches to get I(t) from vHI(t) are possible.

Figure 3 shows that the agreement between the input I(t) function and the derivative of the vHI(t) using the polynomial method is good overall, but clearly has some fluctuations. In order to assess these, we have taken the numerical cosine Fourier transform of the energy spectrum S, with the equivalent counting statistics. Errors were set for each point in the energy spectrum, with the integral of this spectrum being 10^6^ counts as above. Figure 4 shows that for most of the range the error propagation is similar in the two methods, and is probably the best that can be achieved. Similarly, we have used a simple Monte Carlo approach to reduce Gaussian errors during the numerical derivative (Fig. 4), which again gives approximately the same agreement with the input function. We conclude that there is a number of approaches to obtaining the derivative from the “experimental” vHI(t) that reduce the consequences of counting-errors to those of standard methods that require the whole energy spectrum.

## “Experimental” Validation

**Figure 5:**
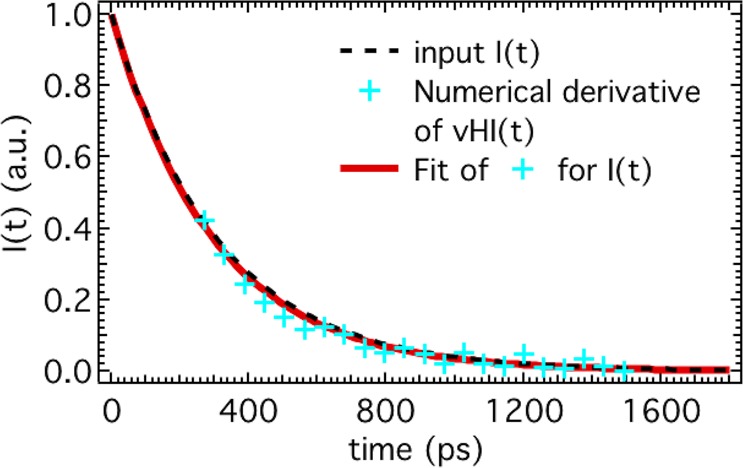
“Experimental” validation by a McStas simulation (of a new instrument designed to access vHI(t) directly). The 25 points (cyan) are the “measured” I(t) obtained by the numerical derivative of the “measured” vHI(t). Each of those 25 points has been measured (i) at 25 different monochromator-to-sample distances corresponding to 25 different value of Δω_R_ (so *t*_*obs*_), (ii) but with fixed value of Δω_F_, as per Eq. (1). The best fit of the “experimental” I(t)-points (red curve) agrees very well with the input I(t) (dashed black curve).

We can make one final step towards a practical instrument by estimating effects of beam inhomogeneities, placement of instrument components, and some account of the scattering processes. Several designs are feasible, but readers are referred to ref.^12^ for details of our recent McStas^27^ instrumental design that we will use in this discussion. This consists of a standard backscattering geometry spectrometer with a stationary focussing monochromator. The distance from the monochromator to the sample is varied, and the focussing is adjusted to maintain the sample at the focal point. This causes the variation of Δω_R_ (so *t*_*obs*_) at the sample. A crystal filter of fixed width Δω_F_ ≪ Δω_R_ selects the narrow range of neutron-counts to be integrated (SM Fig. 5). Two samples were run. Firstly, vanadium which is a purely incoherent scatterer with no measurable processes on the time-scale of interest. This was used to determine the effect of the finite energy-width of the filter, Δω_F_, and other instrumental-errors. Secondly, a sample with a scattering process equivalent to a single exponential decay with tau = 200 ps. Twenty-five incrementally spaced observation-times, *t*_*obs*_, were selected by changing the focal-length of the monochromator. The ratio of the elastic intensity to the total intensity in the scattered beam was determined to obtain vHI(t = *t*_*obs*_). Data-treatment consisted of taking the numerical derivative of the sample vHI(t) and dividing by that of the vanadium. Note that in this case the numerical derivatives were used directly. The results in Fig. 5 illustrate an exponential-decay function that fits the data, in reasonable agreement with the input. For more details please refer to “*SM Paragraph 5*” first, and then to ref.^12^ directly.

## Conclusions and Remarks for the Future

In summary, the van Hove distribution function, I(t), can be used to describe the dynamics of physical systems, and traditionally neutron scattering has accessed this using energy-exchange measurements. However, we show that I(t) can be obtained directly from the proportion of species that have remained static within time *t* (conceptually elastic scattering). More specifically, we have shown that, if a mis-match between the primary and secondary spectrometers is maintained, the elastic neutron-scattering intensity as a function of observation time (i.e. instrumental energy resolution) corresponds to the running-time integral of I(t) (which we denote as van Hove integral vHI(t)). The measured vHI(t) can easily be compared directly with the increasingly common molecular-dynamics simulations, or used as its derivative, I(Q, t).

This new theoretical result has been successfully tested by a series of numerical simulations, and an *in silico* experiment carried out on McStas on an *ad hoc* instrumental design. To distinguish our new method from previous attempts, we refer to it as “Elastic Scattering Spectroscopy, ESS”.

Overall, the energy-exchange and vHI methods are equivalent, but in many instances one will have practical advantages over the other. To date the focus has been entirely on the energy-transfer method so it is likely that there are instances where our vHI method would be better.

At the moment, none of the available neutron spectrometers worldwide can be used to measure vHI(t) and then access I(t) as proposed here. Recently, we proposed *ad hoc* instrument concepts to do so, and we hope these will motivate better new designs that will be built. We believe that our new ESS approach can impact the use of neutron scattering for dynamics in the several cases of study in which either the complexity of the systems and/or their low availability and/or the sample environment requirements make standard approaches for dynamics, as QENS and NSE, difficult. This could be certainly the case for soft matter and bio- systems. Finally, we also believe that extremely simple instrumental designs can be achieved, leading/opening the way to compact versions of the instrument optimized to work for small neutron sources based on radio-frequency quadrupole accelerators (RFQ).

## Supplementary information

Supplementary Material
