# Supplementary material for: Dynamics from elastic neutron-scattering via direct measurement of the running time-integral of the van Hove distribution function
Source: Sci Rep. 2019 Aug 2;9:11284. doi: 10.1038/s41598-019-46835-z (PMC6677729; doi:10.1038/s41598-019-46835-z)
Supplement: Supplementary file 1 — Supplementary Material [file 41598_2019_46835_MOESM1_ESM.pdf]

Supplementary Information for "Dynamics from elastic neutron-scattering via direct measurement of the running time-integral of the van Hove distribution function"

Antonio Benedetto[*1,2,3,4,5] and Gordon J. Kearley*[2]

[1]*School of Physics, University College Dublin, Dublin 4, Ireland*

[2]*School of Chemistry, University College Dublin, Dublin 4, Ireland*

[3]*Conway Institute of Biomolecular and Biomedical Research, University College Dublin, Dublin 4, Ireland*

[4]*Department of Sciences, University of Roma Tre, Rome, Italy*

[5]*Laboratory for Neutron Scattering, Paul Scherrer Institut, Villigen, Switzerland*

Corresponding author: antonio.benedetto@ucd.ie

Supplementary information

This supplementary material (SM) is intended to complete and support the main letter by presenting in more details the theoretical framework, and the data processing protocol.

1. The most general theoretical framework:

Let us consider the most general layout of a neutron scattering experiment: neutrons come from the source via a neutron guide and are energy-shaped in the primary spectrometer. They then hit the sample and are scattered into the analyser of the secondary spectrometer before reaching the detector bank (SM Fig. 1a).

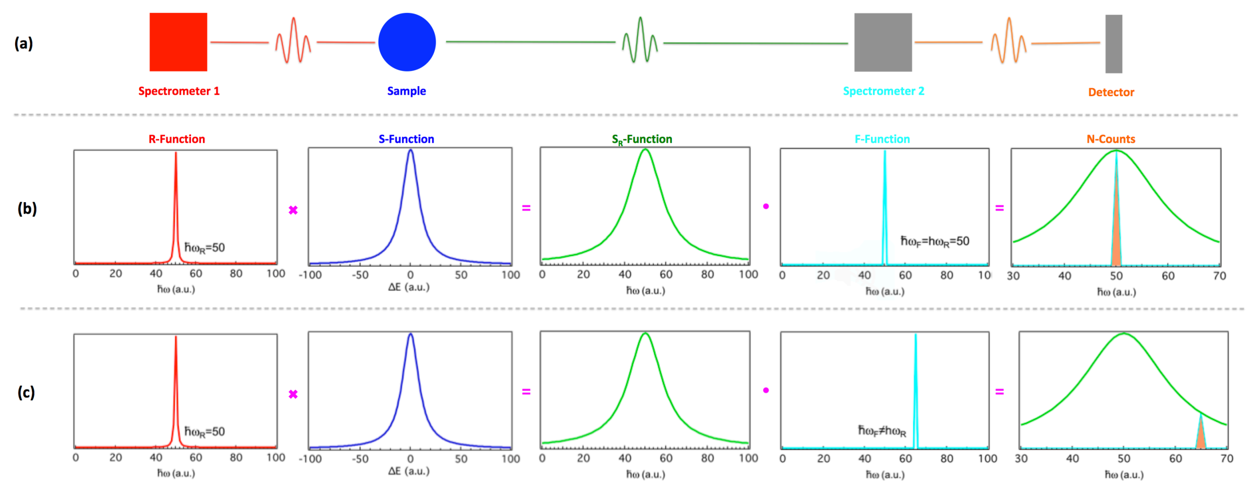

SM Fig. 1 – (a) The most general layout of a neutron scattering experiment in which the interplay between the primary and the secondary spectrometer enables the measurement of either the elastic (b) or the inelastic (c) contribution. Following Brockhouse[2], it is the inelastic scattering that has been routinely used to access system dynamics, this being done by changing either ω_R or ω_F, to measure an energy exchange. We propose a new method to access the system dynamics by collecting the purely elastic contribution as a function of either $\Delta\omega_R$ or $\Delta\omega_F$.

Neutrons from the primary spectrometer can be mathematically described by the distribution function of their energies, $R(\omega;\omega_R,\Delta\omega_R)$, which is often a near symmetric function, centred around a specific energy value, $\hbar\omega_R$, with a variance, $\hbar\Delta\omega_R$. In many cases, this is a Gaussian function but others such as a Lorentzian or triangular function, also exist. The energy integral of R is the total number of neutrons coming from the primary spectrometer that hit the sample.

The sample can be mathematically described as the distribution function of the exchanged energies of the system (atomic and molecular) processes, $S(Q,\Delta E)$, or, by its time Fourier transform $I(Q,t)$ known as intermediate scattering function, or, by its space-time Fourier transform $G(r,t)$ known as the van Hove (total) distribution function. Ignoring the detailed balance, this near symmetric sample-component is centred at $\Delta E=0$, and has a distribution of inelastic events. If present, the symmetric broadening of the elastic component is called quasi-elastic scattering, and its width and intensity are related to the system dynamics. The sample function is a probability distribution that is normalised.

When a neutron with energy $\hbar\omega_R$ hits the sample, it can be scattered by a nucleus of the system atoms, with which it may exchange energy and momentum. Assuming single scattering, the scattered neutron-energy, $\hbar\omega_{NEW}$, is related to a single scattering event in which the energy $\Delta E_{Exchanged}$ passes between the neutron and the system, such as $\hbar\omega_{NEW}=\hbar\omega_R-\Delta E_{Exchanged}$. This energy exchanged corresponds to an allowed energy change of the system. In this respect, S(Q,ΔE) can be seen as the probability distribution that an incoming neutron will exchange an energy ΔE with the system, so changing its energy from $\hbar\omega_R$ to $\hbar\omega_{NEW}=\hbar\omega_R-\Delta E_{Exchanged}$.

After the scattering process, the energy distribution is described by the convolution of the energy distribution incident-beam, $R(\omega;\omega_R,\Delta\omega_R)$, with the distribution of the system-exchanged energies, S(Q,ΔE):

$$S_R(Q,\omega;\omega_R,\Delta\omega_R) = S(Q,\Delta E)\otimes R(\omega;\omega_R,\Delta\omega_R) \tag{1}$$

$S_R(Q,\omega;\omega_R,\Delta\omega_R)$ is referred to as the "scattering law', which is a symmetric function as S(Q,ΔE), but now centred at $\hbar\omega_R$ and with a different width taking into account the spread in the incoming neutron-energies, $\hbar\Delta\omega_R$, which includes the uncertainty of the incident energy. For this reason, R is usually called the energy resolution-function of the (primary) spectrometer. The total area of S_R integrated in energy and space is equal to the number of scattered neutrons. For a perfectly monochromatic incident-beam, $R=\delta(\omega-\omega_R)$, then $S_R=S(Q,\hbar\omega=\Delta E+\hbar\omega_R)$. Clearly, in this case the energy distribution after the scattering process maps the system exchanged energies.

The scattered beam then reaches the secondary spectrometer. Only the neutrons fulfilling the instantaneous time and space conditions of the secondary-spectrometer analysis are detected. For this single measurement (as opposed to a scan that we will discuss later) the secondary spectrometer acts as a filter. If $F(\omega;\omega_F,\Delta\omega_F)$ describes the probability that a neutron with energy $\hbar\omega_F$ passes through this filter, the number of neutrons reaching the detectors is:

$$N_{neutron} = \int_{-\infty}^{\infty} S_R(Q,\omega;\omega_R,\Delta\omega_R)F(\omega;\omega_F,\Delta\omega_F)\,d\omega \tag{2}$$

Usually F is a symmetric function centred around a specific energy value, $\hbar\omega_F$, and with a certain width, $\hbar\Delta\omega_F$. For example this is a delta-function for a perfect crystal analyser, and only the neutrons with the exact matching energy will be detected. The F-function is a probability distribution that is normalised, i.e. its energy integral has to be equal to 1.

By combining Eq. 1 and Eq. 2, and taking into account the normalisations detailed above, the total number of neutron reaching the detector is:

$$N_{neutron} = \frac{\int_{-\infty}^{\infty}[S(Q,\Delta E)\otimes R(\omega;\omega_R,\Delta\omega_R)]\cdot F(\omega;\omega_F,\Delta\omega_F)\,d\omega}{\int_{-\infty}^{\infty}S(Q,\Delta E)d(\Delta E)\cdot\int_{-\infty}^{\infty}F(\omega;\omega_F,\Delta\omega_F)\,d\omega} \quad (3)$$

By using the convolution theorem, and assuming that the two probability distribution-functions are normalized and even, we have:

$$N_{neutron} = \frac{\int_{-\infty}^{\infty}[S(Q,\Delta E)\otimes R(\omega;\omega_R,\Delta\omega_R)]\cdot F(\omega;\omega_F,\Delta\omega_F)\,d\omega}{\int_{-\infty}^{\infty}S(Q,\Delta E)d(\Delta E)\cdot\int_{-\infty}^{\infty}F(\omega;\omega_F,\Delta\omega_F)\,d\omega} =$$

$$= \int_{-\infty}^{\infty}[S(Q,\Delta E)\otimes R(\omega;\omega_R,\Delta\omega_R)]\cdot F(\omega;\omega_F,\Delta\omega_F)\,d\omega =$$

$$= \int_{-\infty}^{\infty}\left[\int_0^{\infty}I(Q,t)R(t;\omega_R,\Delta\omega_R)e^{-i\omega t}\,dt\right]\cdot F(\omega;\omega_F,\Delta\omega_F)\,d\omega =$$

$$= \int_0^{\infty}dt\int_{-\infty}^{\infty}d\omega I(Q,t)R(t;\omega_R,\Delta\omega_R)F(\omega;\omega_F,\Delta\omega_F)e^{-i\omega t} = \quad (4)$$

$$= \int_0^{\infty}I(Q,t)R(t;\omega_R,\Delta\omega_R)\left[\int_{-\infty}^{\infty}F(\omega;\omega_F,\Delta\omega_F)e^{-i\omega t}\,d\omega\right]dt =$$

$$= \int_0^{\infty}I(Q,t)R(t;\omega_R,\Delta\omega_R)F(t;\omega_F,\Delta\omega_F)\,dt \quad (5)$$

Eq. 4 and its integral in energy, i.e. Eq. 5, describe the most general neutron scattering experiment. Eq. 5, in particular, shows the symmetric role played by the first and the second spectrometer for a single measurement. Basically, the number of neutrons reaching the detector banks is unchanged by interchanging the primary and the secondary spectrometers.

SM Fig. 1b and 1c show how Eq. 5 works for the elastic and inelastic scattering, respectively. To show the concept more clearly, the R- and F-functions have been approximated to delta functions. In the purely elastic regime, $\hbar\omega_R=\hbar\omega_F$, only elastically scattered neutrons arrive at the detector (SM Fig. 1b); whereas in the inelastic regime, only the neutrons with energy exchanges $\Delta E=\hbar\omega_R-\hbar\omega_F$ are detected. In the inelastic regime by varying either $\hbar\omega_R$ or $\hbar\omega_F$, it is possible to scan the whole S_R (in green), and then indirectly access S (in blue) by de-convoluting with R or F (in red and cyan, respectively), and I(Q,t) by time-FT. In our method, however, we access the I(Q,t) directly in time domain by operating in the purely elastic regime without any need of FTs and de-convolutions.

In the following we will show in more detail how the standard methods operate and how they can be obtained from our general approach of Eq. 5, and we present our elastic method for dynamics.

2. Our method vs "standard" methods:

2.1 "Standard" methods for dynamics:

As pointed out at the end of the previous paragraph, in the inelastic regime it is possible to access S_R by varying either $\hbar\omega_R$ or $\hbar\omega_F$. This is the method currently used in neutron scattering to access system dynamics, as proposed by Brockhouse for which he received the 1994 Nobel Prize in Physics[2]. Eq. 5 shows the symmetric role played by the primary and the secondary spectrometer for a single measurement. Thus, it is possible to use either a fixed monochromatic neutron-beam and scan the neutron final energies by varying $\hbar\omega_F$ (SM Fig. 2a), or use to vary the distribution of incoming neutrons by varying $\hbar\omega_R$ and detect a specific energy (SM Fig. 2b).

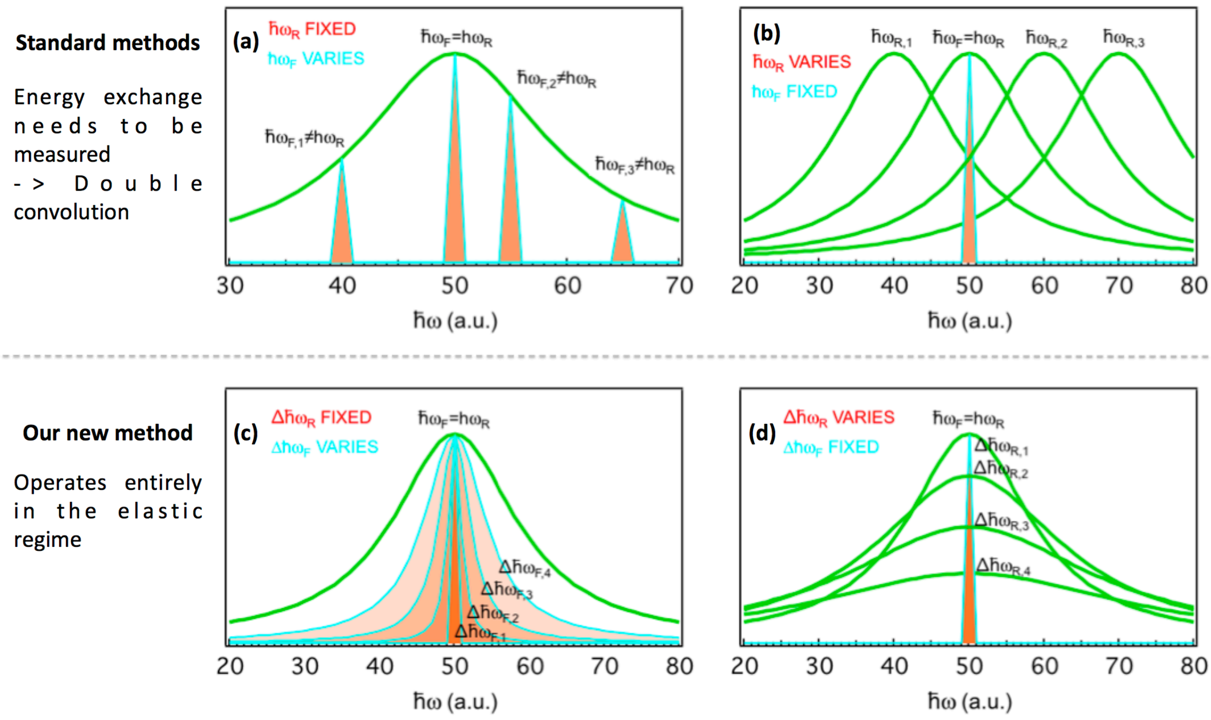

SM Fig. 2 – Comparison between "standard" methods for dynamics (a,b) and our new method (c,d). Standard methods operating in the inelastic regime, need to compute the energy exchange, and result in a double convolution to access S(Q,ΔE). The neutron energy-uncertainties, $\hbar\Delta\omega_R$ and $\hbar\Delta\omega_F$, are usually fixed. Our method is fundamentally different. It operates in the purely elastic regime ($\omega_R=\omega_F$), accessing the running time-integral of the I(t) directly by scanning either $\Delta\omega_R$ or $\Delta\omega_F$. Eq. 5 shows the symmetric role of the two spectrometers: In the standard methods when the energy of the primary spectrometer is fixed, then by varying the energy of the secondary spectrometer it is possible to scan S(Q,ΔE) (a), or when it is the energy of the secondary spectrometer to be fixed, then S(Q,ΔE) can be scanned by changing the energy of the first spectrometer (b). Here the double convolution is apparent. In contrast, our method relaxes one of the energy uncertainties, either $\hbar\Delta\omega_F$ (c) or $\hbar\Delta\omega_R$ (d), with the other being fixed to a narrower value. This accesses the portion of elastic scattering which corresponds to the running-time integral of I(t) as per Eq. 8 and 12, respectively. Case (d) is the one we considered in the main text.

These two protocols are basically equivalent. However, it is easier initially to focusing first on the former, summarised in SM Fig. 2a. The neutrons at the detector have energy-exchange of $\Delta E=\hbar\omega_R-\hbar\omega_F$. By varying $\hbar\omega_F$ and keeping $\hbar\omega_R$ fixed, it is possible to measure the number of neutrons as a function ΔE (see the four peaks in SM Fig. 2a). This number is by definition S_R. Normally the energy uncertainties of the primary and secondary spectrometers are kept similar, i.e. $\hbar\Delta\omega_R\approx\hbar\Delta\omega_F$. From a mathematical point of view, this protocol results in a convolution between S_R and F. Considering that S_R is itself a convolution, S×R, it results in a double convolution (as it is well known). It is easer to see the second convolution in the second symmetric protocol of SM Fig. 2b. In this case, $\hbar\omega_F$ is fixed, and the S_R is scanned by varying $\hbar\omega_R$. This results in energy shifts of S_R being centred at the different $\hbar\omega_R$ (see the different S_R, in green, in SM Fig. 2b). In practice a standard backscattering spectrometer operates exactly in this way. A time modulation of $\hbar\omega_R$ is generated via a doppler device, and after the scattering process, a crystal analyser reflects only the neutrons with a certain energy $\hbar\omega_F$.
We highlight that these standard approaches measure S_R. Then the first step is to de-convolute it with R to get S. This de-convolution process is usually done numerically and by proposing a function that is convoluted with R to obtain S_R. Once a good approximation of S is computed this has to be time-FT to get I(Q,t). There are obvious advantages for methods able to access I(Q,t) without the need of either FTs or de-convolutions, accessing I(Q,t) directly in the time domain.

2.2 Our method for dynamics:
Our approach is based on the consideration that the best way to probe system dynamics is via I(Q,t) directly rather than S_R, and that Eq. 5 contains I(Q,t) already, so we consider the most direct access to I(Q,t) based on Eq. 5.
In our approach we suggest scanning the system dynamics via the species that are at rest within a certain time frame (elastic regime) rather the exchanged energies (inelastic regime) as summarised in Fig. 1 in the main text. Thus, in our proposed set-up $\hbar\omega_R=\hbar\omega_F$, either $\hbar\Delta\omega_R$ or $\hbar\Delta\omega_F$ varies, and Eq. 5 gives:

$$N_{neutron}(Q,\Delta E=0;\Delta\omega_R,\Delta\omega_F)=\int_0^\infty I(Q,t)R(t;\omega_R,\Delta\omega_R)F(t;\omega_R,\Delta\omega_F)\,dt \quad (6)$$

Eq. 6 is the core of our approach, and shows the two symmetric and equivalent ways in which it can be implemented.
One way (given in the main text and summarized in SM Fig. 2d) uses a very narrow filter, i.e. $\hbar\Delta\omega_F<<\hbar\Delta\omega_R$. This means that the F-function in the time domain can be approximated to unity in the time interval in which the R-function is different form zero, giving:

$$N_{neutron}(Q,\Delta E=0;\Delta\omega_R)=\int_0^\infty I(Q,t)R(t;\omega_R,\Delta\omega_R)\,dt \quad (7)$$

Eq. 7 shows that the time integral of I(Q,t) can be scanned via $\Delta\omega_R$ with the primary spectrometer. In the ideal case of a step R-function, it simply corresponds to the definite running-time integral of the van Hove I(Q,t) function up to t_R, i.e. the van Hove integral function vHI($t=t_R$):

$$N_{neutron}(Q,\Delta E=0;t_R)=\int_0^{\infty} I(Q,t)H(t_R-t)\,dt=\int_0^{t_R} I(Q,t)\,dt\equiv vHI(t_R) \tag{8}$$

where t_R is defined as resolution time and is proportional to the inverse of $\Delta\omega_R$. More precisely it is possible to show that (see SM paragraph 2.3):

$$t_R=\frac{1.66}{\Delta\omega_R} \tag{9}$$

As a result, a plot of the derivate of the number of counts against t_R is the van Hove I(Q,t) function:

$$\frac{d}{dt_R}\left\{N_{neutron}(Q,\Delta E=0;t_R)\right\}=\frac{d}{dt_R}\left\{\int_0^{t_R} I(Q,t)\,dt\right\}=I(Q,t) \tag{10}$$

In the main text we have shown numerically and also by an *in-silico* experiment that Eq. 8 holds reasonably well in real cases where the R-function is not a step function. We will also show later in this SM that the condition $\hbar\Delta\omega_F<<\hbar\Delta\omega_R$ allows a partial relaxation of $\Delta\omega_F$ (SM Fig. 3), which has practical implications in improving counting statistics.

In the converse arrangement (summarized in SM Fig. 2c) we use highly monochromated incident beam corresponding to a very narrow R-function. In the condition of $\hbar\Delta\omega_R<<\hbar\Delta\omega_F$, the R-function in the time domain can be approximated to unity during the time interval in which the F-function is non-zero, giving:

$$N_{neutron}(Q,\Delta E=0;\Delta\omega_F)=\int_0^{\infty} I(Q,t)F(t;\omega_R,\Delta\omega_F)\,dt \tag{11}$$

Eq. 11 has the same form of Eq. 7, but we are now scanning the filter width to measure the van Hove integral. Also in this case, with the ideal case of a step function as F-function, it simply corresponds to the definite running-time integral of the van Hove I(Q,t) function up to t_F, i.e. the van Hove integral function vHI($t=t_F$):

$$N_{neutron}(Q,\Delta E=0;t_F)=\int_0^{\infty} I(Q,t)H(t_F-t)\,dt=\int_0^{t_F} I(Q,t)\,dt\equiv vHI(t_F) \tag{12}$$

where t_F is defined as filter time and is proportional to the inverse of $\Delta\omega_F$ as t_R in Eq. 9, i.e. $t_F=1.66/\Delta\omega_F$. Also in this case, a plot of the derivate of the number of counts against t_F is the van Hove I(Q,t) function:

$$\frac{d}{dt_F}\left\{N_{neutron}(Q,\Delta E=0;t_F)\right\}=\frac{d}{dt_F}\left\{\int_0^{t_F} I(Q,t)\,dt\right\}=I(Q,t) \tag{13}$$

In summary, our method is based on the direct measurement of the van Hove integral, vHI(t), by measuring the portion of the system that is moving more slowly than the sensitivity of the experimental set-up by scanning the time range with either $\Delta\omega_R$ or $\Delta\omega_F$. Then the van Hove function I(Q,t) can be extracted by computing the derivative of this integral as described in the main text and in this SM paragraph.

2.3 From the instrumental energy resolution, Δω, to the (instrumental resolution) observation time, t_R:

In this paragraph the relationship between the "instrumental resolution time, t_R", and the "instrumental energy resolution, $\Delta\omega_R$", anticipated in Eq. 9 is derived. The resolution time has been also referred to as "observation time, t_{obs}". We are deriving this relationship for the most common case of Gaussian function as R-function. As mention above, R has to be normalized in the ω-space to the total number of incoming neutrons, N, (Eq. 14):

$$R(\omega,\Delta\omega=1.177\sigma)=\frac{N}{\sqrt{2\pi}\sigma}Exp\left[-\frac{\omega^2}{2\sigma^2}\right]=\frac{N}{\sqrt{2\pi}\,\frac{\Delta\omega}{1.177}}Exp\left[-\frac{\omega^2}{2\left(\frac{\Delta\omega}{1.177}\right)^2}\right] \tag{14}$$

$$\int_{-\infty}^{\infty} R(\omega,\Delta\omega)\,d\omega=N \tag{15}$$

with σ as the variance of the distribution and Δω=1.177σ, as commonly used in experiments, is the HWHM of the distribution. The time-FT of Eq. 14 gives R in the time domain (Eq. 16):

$$R(t;\Delta\omega)=\frac{1}{\sqrt{2\pi}}\int_{-\infty}^{\infty} R(\omega;\Delta\omega)Exp[-i\omega t]\,d\omega=\frac{N\frac{\Delta\omega}{1.177}}{\sqrt{2\pi}}Exp\left[-\frac{t^2\left(\frac{\Delta\omega}{1.177}\right)^2}{2}\right] \tag{16}$$

By re-arranging the argument of the exponential, Eq. 9 linking the energy resolution to the observation time can be eventually determined (Eq. 18):

$$R(t;t_R=1.66/\Delta\omega)=\frac{N\frac{\Delta\omega}{1.177}}{\sqrt{2\pi}}Exp\left[-\left(\frac{t}{\frac{1.66}{\Delta\omega}}\right)^2\right]=\frac{N\frac{\Delta\omega}{1.177}}{\sqrt{2\pi}}Exp\left[-\left(\frac{t}{t_R}\right)^2\right] \tag{17}$$

$$t_R=\frac{1.66}{\Delta\omega} \tag{18}$$

It is useful at this point to remember the transformation factor form *energy* to *time*:

$$\frac{\Delta\omega[\mu eV]}{\Delta\omega[ps^{-1}]} = 657.9\ \mu eV \cdot ps \tag{19}$$

To conclude this paragraph, the same approach as followed above can be used to adapt Eq. 9 to different real cases of R-functions. Moreover, a numerical evaluation of Eq. 8 with the actual resolution function of the particular spectrometer can be used to estimate or possibly calibrate out the discrepancy originated by not using a step function for R (see main text).

2.4 Neutron Spin Echo:

We want to conclude this theoretical framework by showing that Eq. 4 can also be used for determining the neutron spin-echo (NSE) case. NSE is currently used to access system dynamics. However, even though it accesses the I(Q,t) directly, it entails the energy exchanged (via the neutron spin). For this reason we have included NSE in the "standard" approaches.

In NSE the primary energy-resolution is very narrow, this basically means that the R-function can be considered equal to unity in the time interval in which the F-function is different form zero. Moreover, filtering in NSE is done with a polariser filter, giving for the F-function a cosine function, Cos($\omega \cdot t_F$), where t_F is known as Fourier time and depends on instrumental parameters. Finally, NSE is usually used in the echo-condition, which in our framework means $\omega_R=\omega_F$. These inserted into Eq. 4 give back the basic NSE equation, i.e. Eq. 20:

$$N_{neutron} = \frac{\int_0^\infty dt \int_{-\infty}^\infty d\omega I(Q,t) R(t;\omega_R,\Delta\omega_R) F(\omega;\omega_F,\Delta\omega_F) e^{-i\omega t}}{\int_{-\infty}^\infty S(Q,\Delta E) d(\Delta E) \cdot \int_{-\infty}^\infty F(\omega;\omega_F,\Delta\omega_F) d\omega} =$$

$$= \frac{\int_0^\infty dt \int_{-\infty}^\infty d\omega I(Q,t) \cos(\omega \cdot t_F) e^{-i\omega t}}{\int_{-\infty}^\infty S(Q,\Delta E) d(\Delta E)} =$$

$$= \frac{\int_{-\infty}^\infty d\omega \left[\int_0^\infty I(Q,t) e^{-i\omega t} dt \right] \cos(\omega \cdot t_F)}{\int_{-\infty}^\infty S(Q,\Delta E) d(\Delta E)} =$$

$$= \frac{\int_{-\infty}^\infty S(Q,\Delta E = \hbar\omega) \cos(\omega \cdot t_F) d\omega}{\int_{-\infty}^\infty S(Q,\Delta E) d(\Delta E)} =$$

$$= \langle \cos(\omega \cdot t_F) \rangle = I(Q,t_F) \tag{20}$$

In NSE t_F is scanned by changing the magnetic field or the wavelength of the incoming neutrons.

2.5 Final remarks on the theoretical framework:

We conclude that by taking into account both the primary spectrometer R-function and the secondary spectrometer F-function, our theoretical framework is the most general (to the best of our knowledge). It allows us to introduce our method and distinguish it from the standard QENS and NSE approaches. Nevertheless both QENS and NSE can be determined formally in our general framework summarised in Eq. 5.

3. Matching primary resolution-width with analyser width:

In order to show the closest agreement between the input and calculated functions in the numerical simulation the analyser was chosen to have a fixed narrow band-pass, $\Delta\omega_F$, as suggested by the theory. In practice, if a larger difference can either be tolerated or calibrated out, then the width of the band-pass can be increased as some function of the incident energy width, $\Delta\omega_R$. In the limit, the two functions would be matched, and as discussed in the text this provides a final function that is rather different from the input, as illustrated in SM Fig. 3. We show two other cases in the figure where the incident-energy width and the band-pass of the filter decrease together, but with the band-pass width being held at 0.5 and 0.33 of the incident energy-width. In reality, the time required for measurements at longer system-times dominate the total counting time and the possible gains made by relaxing the filter for measurements at lower times are not as great as may have been expected.

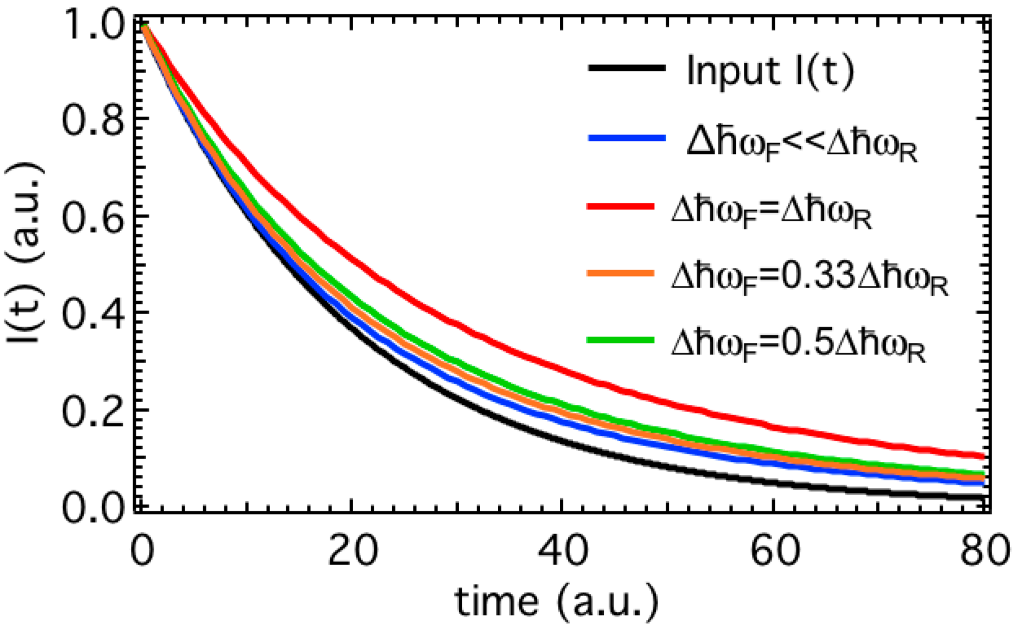

SM Fig. 3 – Effect of changing the energy width of the secondary spectrometer to match the energy width of the primary spectrometer.

4. Data analysis:

In this paragraph more details on the data analysis are provided. SM Fig. 4 summarises the numerical protocol used.

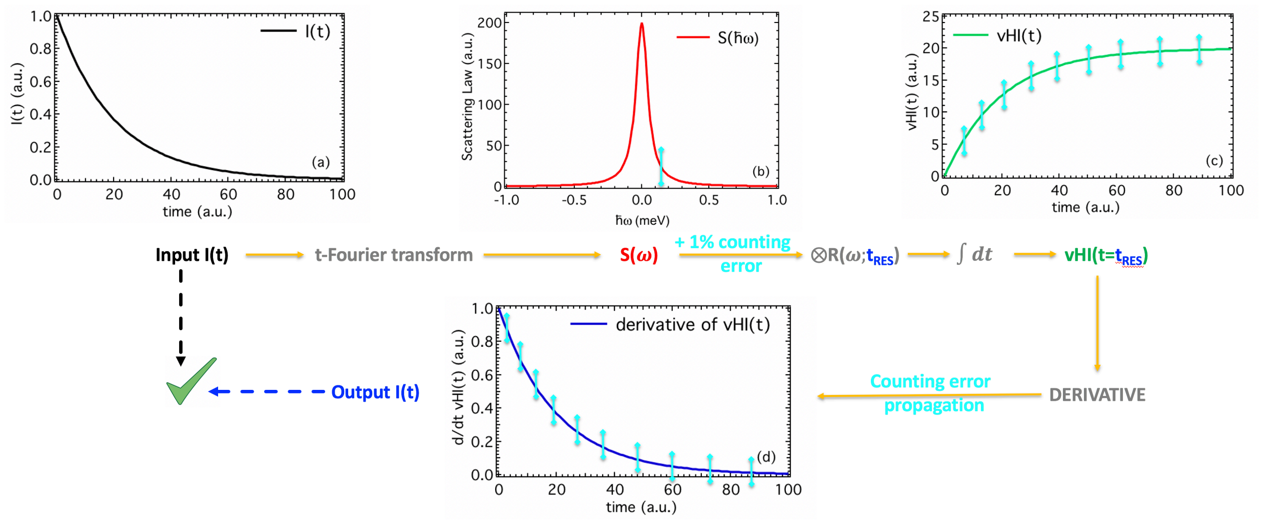

SM Fig. 4 – Summary cartoon of the numerical protocol. The numerical protocol is based on the numerical evaluation of Eq. 3 (main text) over a range of t_{RES} to reproduce an input function from the time domain.

4.1 Polynomial:

In the main text we opted for a polynomial expansion to map the van Hove integral vHI(t) and then extract I(t). Our choice not only provides a convenient route to the derivative, but is also physically meaningful. This is because I(t) can generally be considered as some distribution of exponential functions, for which Taylor expansion is a real vector of the space of the power of time, $\{t^n\}$. As a result, if the vector (a_0, a_1, a_2, a_3, ..., a_N) represents the experimental profile, then the vector (a_1, $2a_2$, $3a_3$, ..., Na_N, 0) is its derivative, i.e. I(t):

$$vHI(t) = \sum_{i=0}^{N} a_i t^i \rightarrow I(t) = \sum_{i=1}^{N} i a_i t^{i-1} \qquad (21)$$

By using a high-order polynomial to represent vHI(t), the coefficients for the polynomial of I(t) are obtained using Eq. 21 (Fig. 3 in the main text).

4.2 Reduction of Gaussian errors:

When the numerical derivative of experimental data with statistical errors is taken, the scatter on the result is much larger. To overcome such difficulty, we mapped the measured function in the vector space of the power of *time* by using the polynomial expansion described in the previous paragraph. Several other methods have been developed by the scientific community to overcome such problems as for example spline. In this paragraph, we present a simple numerical approach we developed to

show how these errors can be reduced whilst taking the derivative. The basic assumption is that the errors on the data are normally distributed so that there must exist a vector of Gaussian-errors that when added to the data removes the scatter. By using a Monte-Carlo approach we try to reduce the scatter on the derivative by adding randomly chosen vectors of Gaussian errors to the experimental function. The cost-function to be minimised has 2 components. Firstly, the sum of the absolute values of the numerically-determined second derivative. The second cost stems from the requirement that the experimental errors are normally distributed, which also requires that width of the scatter between the raw experimental data and the simulated data should be $\sqrt{2\pi}$ multiplied by the height. This cost component is then the difference between the actual width and $\sqrt{2\pi}$. An empirical factor was used to balance the relative weight of these two components. In the main text, Fig. 4 reproduces Fig. 3 together with the derivative of the measured vHI obtained by the Gaussian error approach above-presented.

4.3 Cosine FT of energy spectrum:

The energy spectrum in the numerical simulation was cosine Fourier transformed using the Eq. 20 given for the spin-echo method as a simple consistency check that takes account of the truncation of the energy spectrum.

In the absence of counting errors this produces a smooth curve that agrees well with the input function, so we conclude that the range of our simulated data is adequate for validation purposes (not shown). Counting errors were then introduced on the energy spectrum in a way that is consistent with the errors on the vHI-function and the cosine Fourier transform of this noisy energy spectrum was then made (Fig. 4 in the main text). This results in oscillations in the time-domain function that are broadly comparable with both the function from the polynomial derivative, and the numerical noise-reduction method.

5. Our McStas instrumental designs do measure vHI(t) directly:

In Ref. [12] we have proposed two new instrument designs with the specific purpose to access the elastic neutron scattering intensity as a function of the energy resolution (of the primary spectrometer) directly. One design is suitable for continuous sources and is based on a standard backscattering geometry; the other is suitable for pulsed sources and is based on a time-of-flight option/approach. The former design, which have been used in this letter to “experimentally” validate our new theory, is reported for convenience in SM Fig. 5

This design is very similar to a standard backscattering spectrometer but with a focusing monochromator (as primary spectrometer) able to vary the energy resolution of the neutrons directed into the sample. This is achieved by changing the curvature of the monochromator and adjust accordingly its distance to the sample to maintain the focus. At the longest distance (position A, SM Fig. 5) the monochromator is almost flat, thus a perfect backscattering geometry is restored, and the best energy resolution is achieved (usually order of 1 micro-eV). By increasing the curvature of the

monochromator, and decreasing accordingly its distance to the sample (maintaining the focus) poorer energy resolutions can be achieved, usually down to 100 micro-eV (positions B and C, SM Fig. 5). For instance, the time window of the accessible observation times is largely dictated by the longest and shortest distances available. As a result, the energy resolution is scanned by the first spectrometer, with the other parts of the instrument being identical to standard backscattering spectrometers: that is, the neutrons scattered by the sample do reach the analyzers', and only the "elastic" neutrons will be reflected to the detectors and counted.

The analyzer crystals are matched with the monochromator crystals: the perfect matching is at the fully backscattering condition which is at the longest monochromator-to-sample distance (position A, SM Fig. 5). For all the other distances, the matching is not perfect. This mis-match was perceived as a significant practical disadvantage in Ref. [12], however we now understand that it is vital for Eq. (6) to access I(t). Conventional matching of these widths precludes access to I(t), as reported in SM Fig. 3. As a result, both designs proposed in Ref. [12] can measure vHI(t), but only at the condition that $\Delta\omega_F << \Delta\omega_R$.

For the "experimental" validation presented in this Letter, vHI(t) at twenty-five incrementally spaced observation-times, t_{obs}, were collected by changing the monochromator-to-sample distance from 30 cm corresponding to t_{obs}= 350 ps to 270 cm corresponding to t_{obs}= 1600 ps, At each step the curvature of the monochromator was changed to maintain the focus on the sample, and the filter width was fixed at $\hbar\Delta\omega_F$= 1 micro-eV. The system relaxation time was 200 ps.

Finally, it is important to note the following. The McStas Monte-Carlo simulation of Ref. [12] was originally developed to determine the system overall relaxation time (in complex (bio-) systems) as proposed by the RENS protocol[10,11]. This time corresponds to the time of the inflection point in the elastic scattering intensity versus energy resolution profile: i.e. "there will be an inflection point in the lin-log plot of the elastic intensity versus the instrumental energy-resolution when the energy resolution matches the overall system relaxation-time"[10,11]. As a result, the aim of Ref. [12] was originally to develop new *ad hoc* instrumental designs to measure solely and directly the elastic intensity component of the scattering as a function of the instrumental energy resolution. Generally speaking, this "elastic intensity vs energy resolution" is not vHI(t), and that was not out aim in Ref. [12]. However, thanks to the new theory proposed here, it is now clear that at the condition that $\Delta\omega_F << \Delta\omega_R$, the measured "elastic intensity vs energy resolution" corresponds vHI(t). As a result, the two instrument designs of Ref. [12] can do much more than what was realised when they were designed: they can access the entirely I(t) directly in the time domain by measuring vHI(t). However, this can be achieved only at the condition that $\Delta\omega_F << \Delta\omega_R$, as dictated by Eq. (6). In light of this, the mis-match of the primary resolution and secondary filter-width, is a vital ingredient in the new elastic scattering spectroscopy we are proposing.

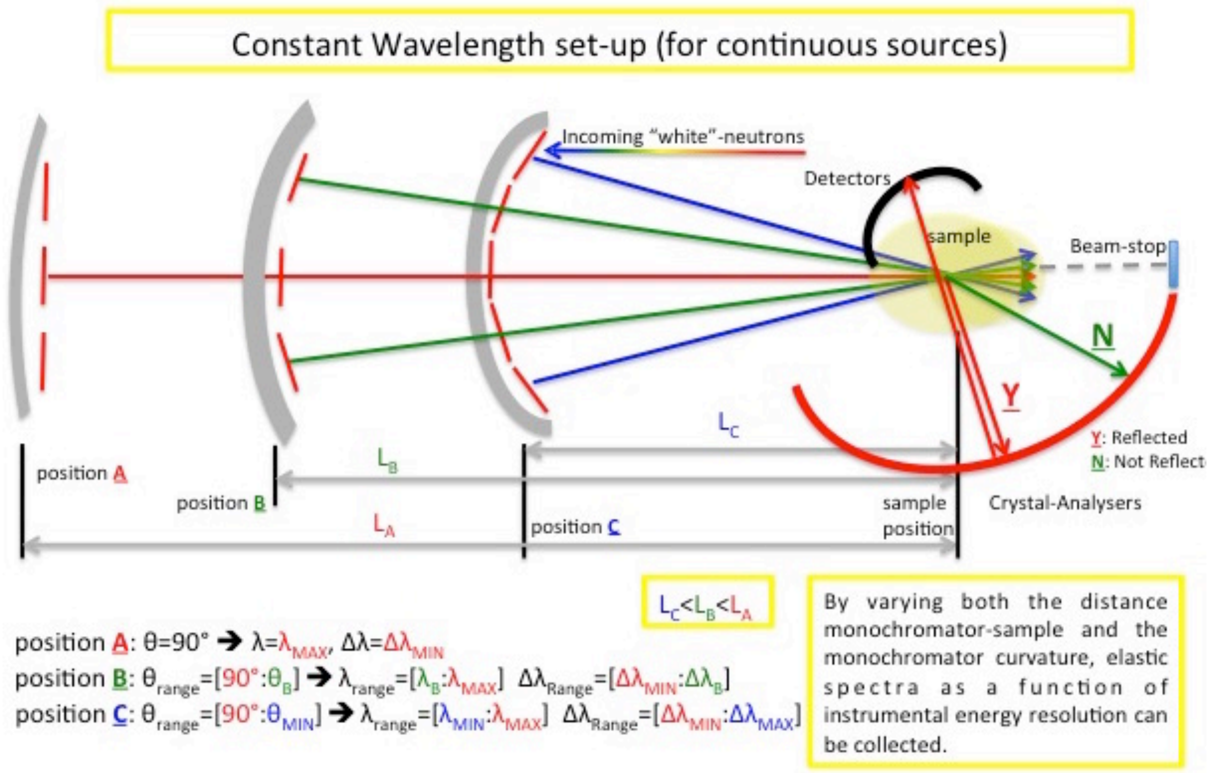

SM Fig. 5 – Lay-out of the spectrometer used in the McStas computation for the "experimental" validation of our new method. The different colours represent different wavelengths, which also have different energy resolutions, different velocities and, in turn, different TOF. Adapted from Ref. [12] and reproduced with the permission from the publisher.
